# Pregnancy outcomes after fetal reduction vs. expectant management of triplet pregnancies: A systematic review and meta-analysis

**DOI:** 10.12669/pjms.42.5.15586

**Published:** 2026-05

**Authors:** Shanshan Lu, Xudong Yang

**Affiliations:** 1 Shanshan Lu, Department of Neonatology, Huzhou Maternity, Child Health Care Hospital, Huzhou, Zhejiang, Province 313000, P.R. China; 2 Xudong Yang, Department of Neonatology, Huzhou Maternity, Child Health Care Hospital, Huzhou, Zhejiang, Province 313000, P.R. China

**Keywords:** Triplet pregnancies, Assisted reproductive technology, Embryo reduction, Miscarriage, Pre-term birth

## Abstract

**Objective:**

This study critically appraises the role of embryo reduction (ER) in managing triplet pregnancies, assessing the risks of miscarriage and pre-term delivery to clarify its strengths and weaknesses.

**Methodology:**

PubMed, Embase, and Scopus were searched to identify relevant studies published up until 31 December 2025. Once identified, a random-effects meta-analysis was performed. Pooled effect sizes were reported as odds ratio (OR) or weighted mean difference (WMD) along with 95% confidence intervals.

**Results:**

Twelve studies were included. The findings from the meta-analysis revealed a significant association between expectant management and pre-term delivery (singleton: OR 0.04, 95%CI:0.02, 0.10; twins: OR 0.25, 95%CI: 0.15, 0.41), and gestational-age (in weeks) (singleton: WMD 5.53, 95%CI: 4.43, 6.64; twins: WMD 2.59, 95%CI: 2.01, 3.18). No significant association was found between ER and risk of miscarriage as well as survival rates until live birth, when compared to expectant management.

**Conclusions:**

ER in triplet pregnancies significantly reduces the risk of pre-term delivery and increases gestational age at birth compared to expectant management. However, ER does not significantly affect the risk of miscarriage or survival rates until live birth.

**Registration No.:** PROSPERO (CRD420251276308).

## INTRODUCTION

Triplet pregnancies have seen a rise in occurrence in recent decades, primarily attributed to two factors: advancing maternal age and the prevalent use of assisted reproductive technology (ART).^1^–^3^ As women delay childbearing, the likelihood of conceiving multiples, including triplets, increases.^4^ Additionally, the advancements in ART techniques, such as in vitro fertilization (IVF), have contributed to the higher incidence of triplet pregnancies.^4^–^6^ The process of transferring multiple embryos during IVF treatment can result in the splitting of one embryo, leading to the formation of triplets with mixed chorionicity.^4^,^7^,^8^

When compared to pregnancies with singletons or twins, triplet pregnancies pose a greater risk of adverse outcomes.^5^,^8^,^9^ Miscarriage, defined as pregnancy loss before 24 weeks of gestation, and pre-term birth, occurring before 34 weeks, are more common in triplet pregnancies.^7^,^10^ These complications can result in significant medical and developmental challenges for both the mother and the infants.^11^–^13^ To mitigate the risks associated with triplet pregnancies, medical interventions have been employed, including a procedure known as embryo reduction (ER).^11^-^14^ ER involves selectively reducing the number of fetuses in the womb, specifically targeting one fetus with a separate placenta or one of the fetuses in a monochorionic pair.^11^,^12^,^14^,^15^ By reducing the number of fetuses, the aim is to improve the overall outcome of the pregnancy and reduce the risks of miscarriage and pre-term birth.^5^,^6^,^10^,^11^

Various techniques are utilized for embryo reduction depending on the chorionicity of the triplets.^4^,^7^,^16^ In tri chorionic tri amniotic (TCTA) pregnancies, where each fetus has its own placenta, ultrasound-guided transabdominal injection of potassium chloride into the fetal heart or chest cavity is commonly used for reduction.^8^,^16^ On the other hand, in dichorionic tri amniotic (DCTA) triplets, which have two fetuses sharing one placenta, ultrasound-guided laser or radiofrequency ablation of the pelvic vessels in one of the monochorionic pair is often employed for reduction.^7^,^8^,^16^

Although embryo reduction has shown positive outcomes in pregnancies with four or more fetuses, its effectiveness in improving obstetric outcomes for triplets reduced to twins or singletons remains uncertain.^8^,^17^ The decision to pursue embryo reduction in triplet pregnancies is a complex one, involving careful consideration of the potential risks and benefits.^8^,^17^ As such, the primary objective of this study is to evaluate the role of ER in managing triplet pregnancies.^1^–^3^,^9^,^13^

This study addresses a significant gap in the existing literature by conducting a systematic review and meta-analysis to compare pregnancy outcomes after fetal reduction vs. expectant management of triplet pregnancies.

## METHODOLOGY

The review was conducted as per PRISMA guidelines.^18^ The study protocol was registered on PROSPERO (CRD420251276308). No protocol amendments were carried out.

### Search Strategy:

A comprehensive search with language restrictions was conducted by two reviewers on the PubMed, Embase, and Scopus databases till 31^st^ December 2025. Details are shown in Supplementary Table 1. The results were screened for relevance using predefined inclusion and exclusion criteria, resulting in the selection of studies aligned with the research objectives. This strategy aimed to gather the most current and pertinent evidence to analyse the associations between these interventions and the specified outcomes.

**Supplementary Table-I:** Search Strategy.

***1. PubMed (MEDLINE)***
((“Pulmonary Embolism”[Mesh] OR “pulmonary embolism”[Title/Abstract] OR “acute pulmonary embolism”[Title/Abstract] OR PE[Title/Abstract]) AND (“Sarcopenia”[Mesh] OR sarcopenia[Title/Abstract] OR “skeletal muscle”[Title/Abstract] OR “muscle mass”[Title/Abstract] OR “muscle area”[Title/Abstract] OR “muscle index”[Title/Abstract] OR psoas[Title/Abstract] OR pectoralis[Title/Abstract]))
***2. Embase***
(‘pulmonary embolism’/exp OR ‘pulmonary embolism’:ti,ab OR ‘acute pulmonary embolism’:ti,ab OR PE:ti,ab) AND (‘sarcopenia’/exp OR sarcopenia:ti,ab OR ‘skeletal muscle’:ti,ab OR ‘muscle mass’:ti,ab OR ‘muscle area’:ti,ab OR ‘muscle index’:ti,ab OR psoas:ti,ab OR pectoralis:ti,ab)
***3. Scopus***
(TITLE-ABS-KEY(“pulmonary embolism” OR “acute pulmonary embolism” OR PE) AND TITLE-ABS-KEY(sarcopenia OR “skeletal muscle” OR “muscle mass” OR “muscle area” OR “muscle index” OR psoas OR pectoralis))
***4. Web of Science***
(TS=(“pulmonary embolism” OR “acute pulmonary embolism” OR PE) AND TS=(sarcopenia OR “skeletal muscle” OR “muscle mass” OR “muscle area” OR “muscle index” OR psoas OR pectoralis))

**Supplementary Table-II:** Risk of bias as per the ROBINS-I methodological tool (low risk of bias: +, high risk of bias: -, lack of clarity: ?).

Study	Confounding bias	Selection bias	Deviation from the intervention	Missing data	Measurement of outcomes	Selective reporting	Classification of the intervention
Cai et al. (2020)	?	?	?	?	?	?	+
Liu et al. (2022)	+	+	+	-	+	+	+
Meng et al. (2022)	+	+	+	+	+	+	+
Balci et al. (2022)	?	?	?	?	?	?	+
Shaw et al. (2021)	+	+	+	-	+	+	+
Sun et al. (2018)	?	?	?	?	?	?	+
Abel et al. (2016)	+	+	+	-	+	+	+
Morlando et al. (2015)	?	?	?	?	?	?	+
Shiva et al. (2014)	+	+	+	-	+	+	+
Chaveeva et al. (2013)	+	+	+	+	+	+	+
Drugan et al. (2013)	+	+	+	+	+	+	+
Antsaklis et al. (2004)	+	?	+	?	+	+	+

### Inclusion Criteria (as per the PECOS criteria):

- ***Population:*** Pregnant women with triplet pregnancies, Both TCTA and DCTA pregnancies.
- ***Exposure:*** ER procedures, including selective reduction of a fetus with a separate placenta or reduction of one of the fetuses in a monochorionic pair or as a singelton.
- ***Comparison:*** Expectant management.
- ***Outcome:*** Obstetric outcomes, including risks of miscarriage (before 24 weeks of gestation), pre-term birth delivery, survival rate, and gestational age.
- ***Study Design:*** Randomized controlled trials (RCTs), cohort studies, case-control studies, and observational studies.

### Exclusion Criteria:

- Studies not focusing on ER in the management of triplet pregnancies.
- Animal studies and in vitro studies.
- Studies not reporting outcomes.
- Studies focusing on pregnancies with more than triplets or different interventions unrelated to ER in triplet pregnancies.

Two reviewers independently evaluated the eligibility of the identified studies. Each study was carefully assessed against predetermined criteria to confirm its relevance to the research question. Disagreements were resolved through discussion and consensus.

### Quality assessment:

The ROBINS-I tool was used to assess potential bias in the cohort trials included in this study, as recommended by Sterne et al.^19^ Two independent reviewers conducted a thorough evaluation of the methodological quality of the included studies. Disagreements were resolved through discussion.

### Data extraction:

Two reviewers independently extracted information regarding study type, groups involved, sample size, and average maternal age. Additionally, we collected data on obstetric outcomes, including rates of miscarriage, gestational age in weeks, pre-term birth, and survival rates. The authors contacted the authors once for missing data via email. If no response was received the study was not included in the meta-analysis.

### Data analysis:

To conduct the data analysis, we utilized Stata version 15.0 and applied a random-effects model.^20^ A meta-analysis was performed to examine the association between fetal reduction (singleton and twins) and expectant management of triplet pregnancy. Continuous outcomes were pooled to calculate weight mean difference (WMD), and dichotomous outcomes were pooled as the odds ratio (OR) with 95% confidence intervals (CI).To evaluate heterogeneity among studies, we used I2 values. I2 values between 0-25% indicated negligible heterogeneity, 25-75% indicated moderate heterogeneity, and ≥75% indicated substantial heterogeneity, following the guidelines of.^21^ Publication bias was assessed using Egger’s test. Sensitivity analysis was conducted removing one study at a time.

## RESULTS

**Fig.1:**
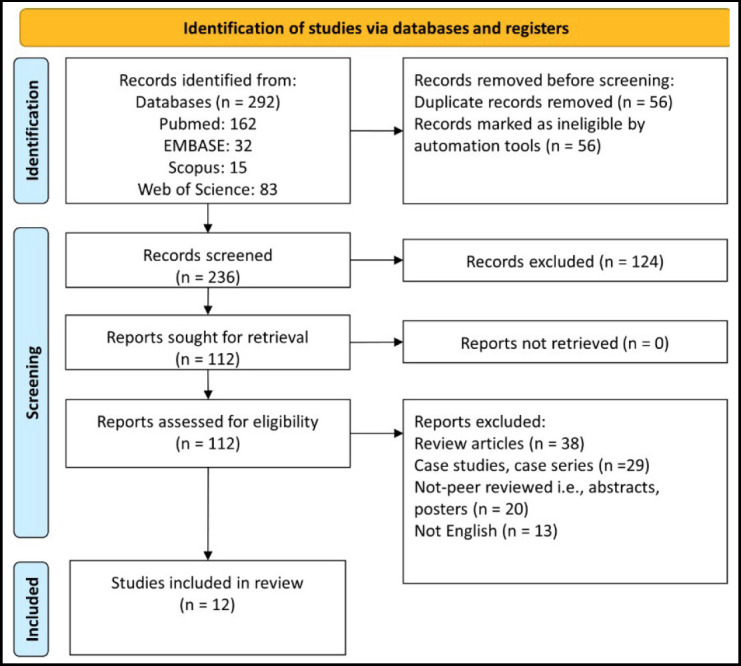
PRISMA flowchart.

PRISMA flowchart is shown in Fig.1. After applying the predetermined inclusion criteria, 12 studies were included. These selected studies exhibited heterogeneity in terms of their designs, with one being a prospective cohort studies^2^ and eleven being retrospective cohort studies.^1^,^6^–^12^,^14^,^16^,^17^ The extracted data from these studies are presented in detail in Table-I.

**Table-I:** Details of included studies for meta-analysis.

Study	Design	Country	Groups and sample size	Method of conception and type of pregnancies	Maternal age (M ± SD years)	Method of reduction
Cai et al. (2020)	RCS	China	RS (N: 84) RT (N: 149) Ex (N: 65)	IVF-Embryo transfer: 298 Dichorionic Tri-amniotic pregnancies	RS (29.6 ± 4.2) RT (29.4 ± 3.9) Ex (28.4 ± 3.7)	KCL
Liu et al. (2022)	RCS	China	RS (N: 76) RT (N: 18) Ex (N: 34)	ICSI: 47 IVF: 81 Dichorionic Tri-amniotic pregnancie	RS (29.4 ± 4) RT (30.3 ± 4.3) Ex (29.2 ± 4.3)	Puncture and aspiration with transvaginal ultrasound
Meng et al. (2022)	RCS	China	RT (N: 24) Ex (N: 19)	IVF: 6 Spontaneous: 37 Monochorionic Tri-amniotic pregnancies	RT (30 ± 0.83) Ex (30 ± 1.0)	RF
Balci et al. (2022)	RCS	Turkey	RT (N: 106) Ex (N: 45)	IVF: 131 Ovulation induction: 14 Spontaneous: 6 Tri-chorionic Tri-amniotic pregnancies	RT (30 ± 8) Ex (29.5 ± 8)	KCL
Shaw et al. (2021)	RCS	UK	RS (N: 9) RT-I (N: 14) RT-II (N: 5) Ex (N: 55)	Artificial reproductive technology: 83 Dichorionic Tri-amniotic pregnancies	RS (35 ± 5) RT-I (33 ± 5) RT-II (39 ± 8) Ex (35 ± 6)	RS: KCL RT-I: RF RT-II: Infrafetal laser
Sun et al. (2018)	PCS	China	RS (N: 14) RT (N: 14) Ex (N: 19)	IVF: 25 Ovulation induction: 2 Spontaneous: 17 ICSI: 3 Dichorionic Tri-amniotic pregnancies	31 ± 4	RS: KCL RT: RF
Abel et al. (2016)	RCS	Germany	RS (N: 30) Ex (N: 16)	IVF: 38 Ovulation induction: 2 Spontaneous: 7 Monochorionic Tri-amniotic pregnancies	RS (34.1 ± 5.3) Ex (34 ± 4.7)	KCL
Morlando et al. (2015)	RCS	UK	RS (N: 10) RT (N: 13) Ex (N: 77)	Natural: 20 IVF/ICSI: 65 Ovulation induction: 1 Unknown: 13 Dichorionic Tri-amniotic pregnancies	Not provided	Not provided
Shiva et al. (2014)	RCS	Iran	RT (N: 57) Ex (N: 58)	IVF/ICSI: 85 IUI: 30 Tri-chorionic pregnancies	RT (31.1 ± 4.8) Ex (29.7 ± 3.7)	KCL
Cha-veeva et al. (2013)	RCS	UK	RS (N: 29) RT (N: 15) Ex (N: 123)	IVF: 137 Ovulation induction: 7 Spontaneous: 23 Dichorionic Tri-amniotic pregnancies	RS (35.3± 3.8) RT (36.5±1.5) Ex (34.3±4.2)	KCL
Drugan et al. (2013)	RCS	Israel	RT (N: 43) Ex (N: 34)	IVF: 30 Ovulation induction: 52 Not provided	RT (30.3 ± 5.4) Ex (30.7 ± 5.3)	KCL
Antsaklis et al. (2004)	RCS	Gre-ece	RT (N: 185) Ex (N: 70)	Not provided	Age ranged from 20-47 years (median of 32 years)	KCL

RS: Reduction to singleton, RT: reduction to twins, Ex: managed expectantly, IVF: In vitro fertilization, ICSI: Intracytoplasmic sperm injection, RF: Radiofrequency ablation, IUI: Intrauterine insemination, KCL: Ultrasound-guided intrathoracic injection of potassium chloride, RCS: Retrospective cohort study, PCS: Prospective cohort study.

### Meta-Analysis outcome:

### Association of fetal reduction and expectant management with a survival rate:

**Fig.2:**
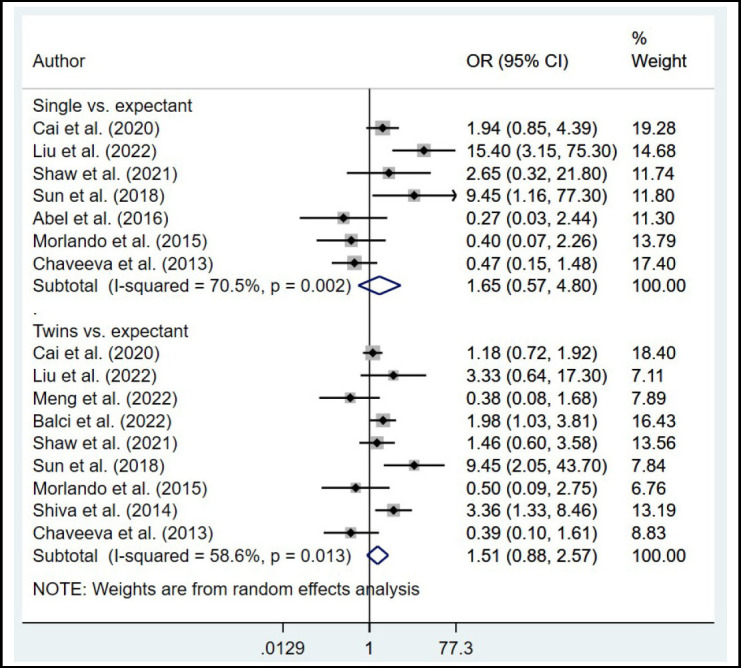
Comparison between foetal reduction and expectant triplet pregnancy for “survival” as the outcome of interest.

Our analysis of seven cohort studies (Fig.2) indicates similar survival rate until livebirth for both the groups i.e., those receiving triplet reduction to a single fetus and those receiving expectant management (OR: 1.65, 95% CI: 0.57, 4.80), with moderate heterogeneity (I^2^: 70.5%). Similarly, the analysis of nine cohort studies (Fig.2) indicates that the survival rate for the group receiving reduction to twins was comparable to the group receiving expectant management (OR: 1.51, 95% CI: 0.88, 2.57), with moderate heterogeneity (I^2^: 58.6%).

### Association of fetal reduction and expectant management with miscarriage:

**Fig.3:**
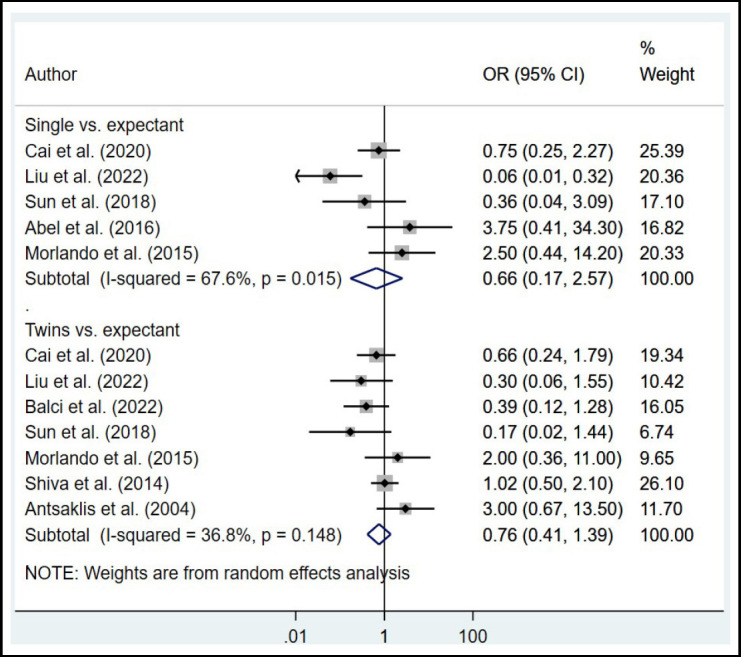
Comparison between foetal reduction and expectant triplet pregnancy for “miscarriage” as the outcome of interest.

Our analysis of five cohort studies (Fig.3) indicates statistically similar risk of miscarriage for the group receiving triplet reduction to a single fetus, compared to the group receiving expectant management (OR: 0.66, 95% CI: 0.17, 2.57), with moderate heterogeneity (I^2^: 67.6%). Similarly, the analysis of seven cohort studies (Fig.3) indicates similar risk of miscarriage between the group receiving triplet reduction to twins and the group receiving expectant management (OR: 0.76, 95% CI: 0.41, 1.39), with moderate heterogeneity (I^2^: 36.8%).

### Association of fetal reduction and expectant management with pre-term birth:

**Supplementary Fig.1:**
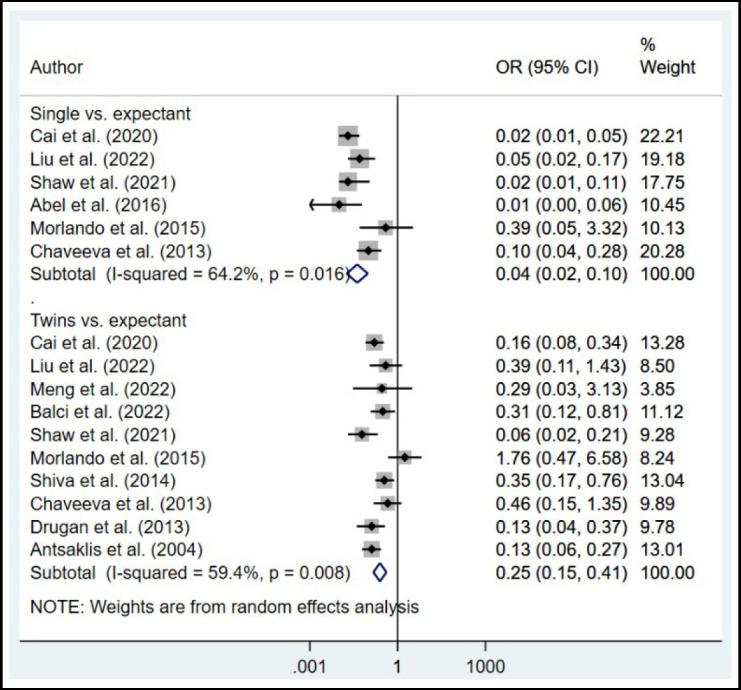
Comparison between foetal reduction and expectant triplet pregnancy for “preterm birth” as the outcome of interest.

Our analysis of six cohort studies (Supplementary Fig.1) indicates a significant decrease in the risk of pre-term birth for the group receiving triplet reduction to a single fetus, compared to those receiving expectant management (OR: 0.04, 95% CI: 0.02, 0.10), with moderate heterogeneity (I^2^: 64.2%). Similarly, the analysis of ten cohort studies indicates a significant decrease in the risk of pre-term delivery for the group receiving triplet reduction to twins, compared to those receiving expectant management (OR: 0.25, 95% CI: 0.15, 0.41), with moderate heterogeneity (I^2^: 59.4%).

### Association of fetal reduction and expectant management with gestational age:

**Supplementary Fig.2:**
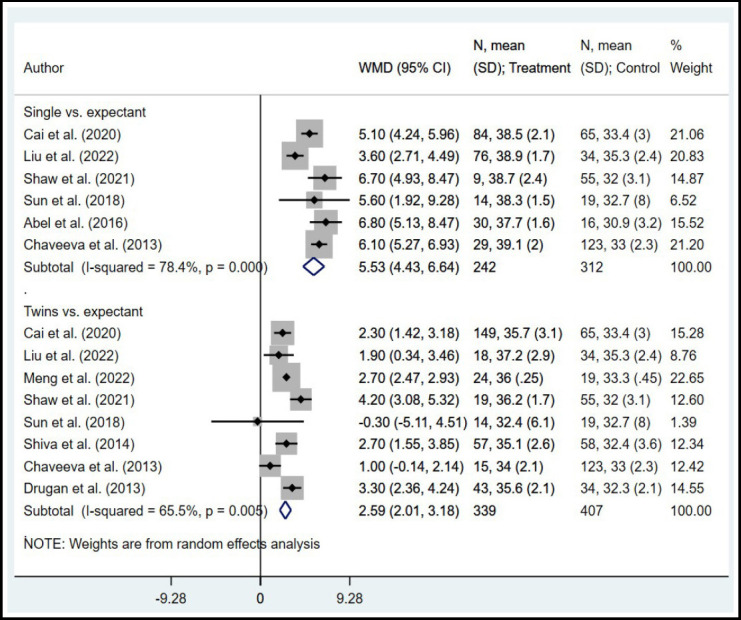
Comparison between foetal reduction and expectant triplet pregnancy for “gestational age (weeks)” as the outcome of interest.

Our analysis (Supplementary Fig.2) indicates significant increase in gestational age (in weeks) among those receiving triplet reduction to single fetus (WMD: 5.53, 95% CI: 4.43, 6.64; N=6, I^2^ = 78.4%) and in those receiving triplet reduction to twins (WMD: 2.59, 95% CI: 2.01, 3.18; N=8, I^2^ = 65.5%), when compared to those receiving expectant management.

### Publication bias & Sensitivity analysis:

Egger’s test did not demonstrate publication bias for any of the outcomes (p>0.05). The results of all outcomes were stable on sensitivity analysis.

### Assessment of Study Quality:

The findings of this assessment are summarized in Supplementary Table-I, revealing a high risk of bias across the included studies. However, several studies had missing data and showed signs of selection bias.

## DISCUSSION

This showed that fetal reduction, whether to singleton or twin gestation, is consistently associated with a significant reduction in preterm birth risk and a notable extension of gestational age at delivery. Conversely, fetal reduction did not significantly impact the rates of miscarriage or the probability of survival to live birth when compared with expectant management.

The pronounced decrease in preterm birth risk following fetal reduction can be attributed to well-established physiological mechanisms linked to the reduction in fetal number. Triplet gestations are characterized by significant uterine overdistension, increased cervical strain, and elevated inflammatory and hormonal activity, all of which contribute to spontaneous preterm labor.^10^,^12^ Fetal reduction mitigates these factors by decreasing uterine stretch and placental competition, thereby enhancing uteroplacental perfusion and reducing mechanical and biochemical stimuli that trigger early labor.^14^ Moreover, the increase in gestational age among pregnancies that underwent reduction is clinically significant. Even modest prolongation of pregnancy by several weeks can markedly reduce neonatal morbidity, including respiratory distress syndrome, intraventricular hemorrhage, necrotizing enterocolitis, and long-term neurodevelopmental impairments.^10^,^12^,^17^ The analysis indicates that reduction to singleton pregnancy resulted in the most significant prolongation of gestation, followed by reduction to twins, suggesting a dose–response relationship between fetal number and gestational duration.

Fetal reduction did not significantly alter the risk of spontaneous miscarriage or the likelihood of survival to live birth when compared with expectant management. This observation implies that early pregnancy loss in triplet pregnancies may predominantly result from factors not substantially influenced by reduction procedures, such as early placental dysfunction, chromosomal anomalies, implantation-related issues, or inherent embryonic viability.^14^,^17^ Although fetal reduction reduces the fetal burden, the procedure itself entails a modest yet definitive risk of pregnancy loss attributable to the intervention, which may negate any potential decrease in spontaneous miscarriage associated with reduced fetal count.^7^ Furthermore, the similarity in survival rates to live birth between reduced and expectantly managed triplet pregnancies suggests that many triplet gestations, despite their elevated risk for extreme prematurity, can still culminate in live births.^14^,^17^ Nevertheless, survival alone does not capture the full clinical burden of triplet pregnancies. Expectantly managed triplet pregnancies may achieve comparable survival outcomes but often at the expense of very early delivery, extended neonatal intensive care, and increased risk of severe short- and long-term morbidity.^10^,^12^ Consequently, the lack of a survival benefit with fetal reduction should not be interpreted as a lack of clinical advantage; rather, it indicates that the primary benefit of reduction lies in enhancing the quality and maturity of survival outcomes through gestational prolongation, rather than in elevating the overall probability of live birth.

Chorionicity is a critical determinant of both baseline risk and the potential benefits and hazards of fetal reduction in triplet pregnancies.^16^ Monochorionic components are associated with unique complications, including twin–twin transfusion syndrome, selective fetal growth restriction, and shared placental vascular anastomoses, which increase the risks of fetal demise and neurological injury irrespective of management strategy.^11^ In such pregnancies, reduction techniques that interrupt placental circulation are often required and may carry different procedure-related risks compared with potassium chloride injection used in trichorionic gestations.^22^

Conversely, trichorionic and dichorionic triplets are primarily threatened by uterine overdistension and placental competition, mechanisms that are more directly ameliorated by reducing fetal number and may explain the more consistent prolongation of gestation observed in these subgroups. The predominance of dichorionic triamniotic pregnancies in the available literature therefore limits the generalizability of pooled estimates to monochorionic triplets, in whom both the natural history and the risk–benefit profile of fetal reduction differ substantially.^16^ Future studies stratified by chorionicity are essential to clarify the variable risks.

Fetal reduction in triplet pregnancies presents intricate ethical and psychosocial considerations that surpass purely clinical concerns. The choice to reduce one or more fetuses encompasses moral, cultural, and personal values and may evoke substantial emotional responses such as distress, guilt, grief, or ambivalence among parents, even when the intervention is medically indicated.^23^ Some couples may view fetal reduction as conflicting with their ethical or religious convictions, whereas others may feel pressured to opt for reduction due to medical advice or concerns about adverse outcomes.^24^ The psychological repercussions can endure well beyond the pregnancy, particularly in cases where parents grapple with feelings of loss or uncertainty regarding the adequacy of their decision.^25^ Therefore, counseling should be thorough, non-directive, and multidisciplinary, involving obstetricians, fetal medicine specialists, and mental health experts whenever possible. Parents need clear information about the medical risks and benefits and should be given time to consider their emotional, ethical, and social factors. This approach ensures that the final decision is well-informed, respects individual autonomy, and aligns with personal values and circumstances.

### Limitations:

Several limitations should be considered when interpreting this review’s findings. Most of the included studies were retrospective cohort analyses, which are prone to selection bias, residual confounding, and incomplete data reporting. There may be differences between women who underwent fetal reduction and those who had expectant management in terms of baseline risk factors, fertility history, chorionicity, and access to specialized healthcare. Additionally, many studies only provided unadjusted estimates, limiting causal interpretations. There was also notable clinical and methodological heterogeneity due to variations in study design, sample sizes, gestational age at reduction, reduction techniques, and outcome definitions, warranting caution in interpreting the results. The ability to perform subgroup analyses based on key factors, such as chorionicity, conception method, and reduction technique, was limited by inconsistent or missing data, making it difficult to identify populations that might benefit most or face higher risks. Moreover, critical maternal and neonatal outcomes, including maternal complications, neonatal morbidity, and long-term neurodevelopmental results, were not reported in sufficient detail, restricting a comprehensive assessment beyond survival rates and gestational age at birth. Additionally, the lack of RCTs and the predominance of data from specific regions and healthcare settings reduce the relevance and applicability of these findings to the broader population of triplet pregnancies. Lastly, while reductions to singleton and twin gestations were examined together, they are clinically different strategies with unique risk–benefit profiles. Reducing to a singleton led to a longer gestation period, whereas reducing to twins may offer a balance between procedural risks and keeping multiple fetuses. As a result, these strategies should not be seen as interchangeable, and clinical decisions should account for the distinct implications of each approach.

This study offers a thorough and current overview of evidence regarding fetal reduction in triplet pregnancies, including a sizable pooled sample and data on both singleton and twin reduction groups. Using a random-effects model and following PRISMA guidelines strengthens the validity and dependability of the results. Nevertheless, more well-structured prospective studies are necessary, especially those that stratify by chorionicity and employ standardized outcome definitions.

## CONCLUSION

Evidence suggests that expectant management is associated with poorer obstetric outcomes, including pre-term birth and shorter gestational age. Elective reduction may have a potential advantage in reducing risk of preterm birth and increasing the gestational age, although further research is needed to establish a conclusive relationship.

### Recommendations:

Future investigations should also address maternal health, long-term neonatal outcomes, and diverse population groups to enhance the applicability of findings.

### Author’s contributions:

**SL:** Literature search, study design and manuscript writing.

**SL and XY:** Data collection, data analysis and interpretation. Critical Review.

**SL:** Manuscript revision and validation and is responsible for the integrity of the study.

All authors have read and approved the final manuscript.
